# Star-Shaped Thermoplastic Elastomers Prepared via RAFT Polymerization

**DOI:** 10.3390/polym15092002

**Published:** 2023-04-23

**Authors:** Hao Ge, Wencheng Shi, Chen He, Anchao Feng, San H. Thang

**Affiliations:** 1 State Key Laboratory of Organic-Inorganic Composites, Beijing University of Chemical Technology, Beijing 100029, China; 2 Center of Advanced Elastomer Materials, College of Materials Science and Engineering, Beijing University of Chemical Technology, Beijing 100029, China; 3 Aerospace Research Institute of Materials & Processing Technology, Beijing 100076, China; 4 School of Chemistry, Monash University, Clayton, VIC 3800, Australia

**Keywords:** thermoplastic elastomers, RAFT polymerization, SIS, star polymer

## Abstract

Styrene-based thermoplastic elastomers (TPEs) demonstrate excellent overall performance and account for the largest industrial output. The traditional methods of preparation styrene-based thermoplastic elastomers mainly focused on anionic polymerization, and strict equipment conditions were required. In recent years, controlled/living radical polymerization (CRP) has developed rapidly, enabling the synthesis of polymers with various complex topologies while controlling their molecular weight. Herein, a series of core crosslinked star-shaped poly(styrene-*b*-isoprene-*b*-styrene)s (SISs) was synthesized for the first time via reversible addition–fragmentation chain transfer (RAFT) polymerization. Meanwhile, linear triblock SISs with a similar molecular weight were synthesized as a control. We achieved not only the controlled/living radical polymerization of isoprene but also investigated the factors influencing the star-forming process. By testing the mechanical and thermal properties and characterizing the microscopic fractional phase structure, we found that both the linear and star-shaped SISs possessed good tensile properties and a certain phase separation structure, demonstrating the characteristics of thermoplastic elastomers.

## 1. Introduction

As a good substitute for rubber, thermoplastic elastomer (TPE) material has been developed vigorously since its commercialization in 1960. It is mainly represented by an ABA-type styrenic block copolymer obtained via anionic active polymerization with the monomer of butadiene or isoprene, and is still the most important TPE product at present [1,2,3]. Suitable TPE components must be chosen in order to obtain a better phase separation structure. Rubber component B is used as a crosslinking bridge to connect different phase regions of plastic A so as to achieve the overall physical crosslinking effect [3].

For a synthetic method, however, traditional anionic polymerization has a weak resistance to water and oxygen, thereby requiring more demanding conditions and equipment [4,5,6]. In addition to traditional anionic polymerization, researchers have developed a series of methods such as ring-opening polymerization (ROP) [7,8,9,10], active cation polymerization [11], click chemistry [12,13] and others to synthesize or modify thermoplastic elastomers and obtain products with different functions. With the rapid development of active polymerization in the past 20 years, these methods aim to promote more moderate reaction conditions for thermoplastic elastomer composites.

Among these methods, controlled/living radical polymerization (CRP), which has been widely developed in recent years, includes atom transfer radical polymerization (ATRP), nitroxide mediated radical polymerization (NMP) and reversible addition–fragmentation chain transfer (RAFT) [14]. CRP can be applied to almost all vinyl monomers by means of free radical mechanism synthesis, and its characteristics of active polymerization give it an excellent molecular design ability [15,16,17]. Thus, it is natural for researchers to attempt to gradually apply it to the large-scale production of rubber and plastics.

The ATRP method was applied earlier in the synthesis of related fields [15,16,18,19,20,21,22,23,24]. Recently, Kurokawa et al. used polymethyl methacrylate (PMEA) and polymethyl methacrylate (PMMA) as monomers to prepare PMMA-PMEA-PMMA thermoplastic elastomers with different PMMA volume fractions (*f*_MMA_). When the *f*_MMA_ was 0.40, the triblock copolymer showed good antithrombogenicity performance, which may have been related to the microphase separation structure of the copolymer detected by AFM [25]. The NMP method has also been applied in related fields. Using the difunctional initiator DEPN_2_ and a phosphorus ionic liquid monomer as raw materials, Cheng et al. synthesized a poly(St-*b*-nBA-*b*-St) (SBAS) ABA-type triblock copolymer containing phosphoric cation. When phosphine units (BPCl and OPCl) were added to the outer block, the phosphoric cation promoted the self-assembly of amphiphilic phosphine units in the block copolymer system, resulting in a well-defined microphase and tunable flow behavior [26]. RAFT polymerization can be carried out under milder conditions while maintaining a narrow molecular weight distribution and designing molecular structures [27,28,29]. Therefore, the RAFT method has been extensively studied in the past few years [17,30,31,32,33,34,35]. Luo and his co-workers have studied emulsion polymerization for the preparation of thermoplastic elastomers for years [36,37,38,39,40]. They synthesized a triblock copolymer SBAS film which could be used as a dielectric elastomer to fabricate soft robotics or fiber actuators due to its low Young’s modulus and high dielectric constant [41,42,43,44,45,46].

Controlled/living radical polymerization is particularly useful in the field of thermoplastic elastomers because of its ability to design molecular structures in addition to synthesizing polymers with complex components. It is well-known that the properties of thermoplastic elastomers are derived from their physical crosslinking characteristics. In addition to the modifications of components or blending, designing branched or star molecules is a relatively low-cost and efficient method of enhancing the strength of elastomers [47,48,49]. When linear SBSs were first designed, star SBSs were also designed and showed good mechanical strengths at a similar molecular weight. This is because a single crosslinking point does not greatly affect the flow performance of molecules but acts as an anchor point to enhance the binding force between separated phases. There are usually three methods of synthesizing star polymers: the graft-onto, graft-from and arm-first methods. Compared with the other two methods, the arm-first method allows for better control over the molecular weight distribution, as the arm polymer is synthesized first with a higher star-forming efficiency. The star polymers synthesized via the arm-first method are called core crosslinked star polymers [50,51,52].

Unfortunately, there are no precedents of star-shaped thermoplastic elastomers synthesized via the arm-first method; nor are there reports on the mechanical properties of core crosslinked thermoplastic elastomers. Thus, using the arm-first method, we achieved the successful synthesis of star molecules from SIS in thermoplastic elastomers via RAFT polymerization and characterized their properties as another reference for applying the CRP method to the synthesis of elastomers.

## 2. Materials and Methods

### 2.1. Materials

Styrene (St, 99%), isoprene (Ip, 99%) and a divinylbenzene (DVB, 98%) crosslinker were passed through an alkaline alumina column to remove inhibitors. The 2,2’-Azobis(2-methylpropionitrile) (AIBN, 98%), di-tert-butyl peroxide (DTBP, 99%), 2-sec-butyl-4,6-dinitrophenol (DNBP), toluene (AR) and *N*,*N*-Dimethylformamide (DMF, AR) were used without further purification. The synthesis and purification of the small RAFT agent, 4-cyano-4-[(dodecylsulfanylthiocarbonyl)sulfanyl] pentanoic acid (DTTCP), are shown in the Supporting Information.

### 2.2. Sample Preparation

#### 2.2.1. Synthesis of Poly(styrene-*b*-isoprene) (PS-*b*-PI) Diblock Copolymer

Firstly, the macro-RAFT agent PS-CTA was synthesized using styrene as monomer: DTTCP (1 g, 2.5 mmol), St (15.5 g, 148 mmol), AIBN (40.6 mg, 0.25 mmol), and 16.5 g of DMF were introduced into a 50 mL Schlenk flask. After three cycles of freezing–vacuuming–dissolution, the dissolved oxygen in the system was completely removed, and the flask was placed into an oil path at 70 °C for 12 h. The mixture was precipitated in methanol three times, and the product was collected from the sediment in a vacuum-drying oven.

Then, the diblock copolymer PS-*b*-PI was synthesized using isoprene as monomer: PS-CTA (10 g, 2.5 mmol), Ip (67.61 g, 1 mol), DTBP (0.12 g, 0.83 mmol) and 16 g of toluene were added into a 250 mL spherical Schlenk flask. The vacuuming–freezing–dissolution operation was the same as above, and the solution was reacted at 115 °C for 48 h. After precipitation and drying, the purified PS-*b*-PI was analyzed via ^1^H NMR and GPC.

#### 2.2.2. Synthesis of Poly(styrene-*b*-isoprene-*b*-styrene) (SIS) Linear Triblock Copolymer

Using St as monomer and PS-*b*-PI as macro-RAFT agent, a similar procedure was employed for the polymerization of the linear triblock copolymer PS-*b*-PI-*b*-PS (SIS), as above. After purification, the product was analyzed via ^1^H NMR and GPC.

#### 2.2.3. Synthesis of Poly(styrene-*b*-isoprene-*b*-styrene) (SIS) Star Polymer

The SIS star polymer was fabricated by using the “arm-first” strategy, with PS-*b*-PI as the molecular arm. Take entry 30 in Table S1 as an example: AIBN (0.059 g, 0.36 mmol) was taken in toluene (10 mL, 8.66 g); then, 100 μL of the solution was pipetted and added to the mixture of PS-*b*-PI-CTA (0.5 g, 0.036 mmol), St (0.13 g, 1.28 mmol), DVB (0.15 g, 0.86 mmol) and toluene (2.4 mL, 2.09 g) in a 10 mL Schlenk flask. The vacuuming–freezing–dissolution operation was the same as above, and the solution was reacted at 70 °C for 48 h. Then, 2000 ppm of DNBP was added to the reaction solution immediately after the reaction to suppress the effect of the residual crosslinker. The purified SIS star polymer was analyzed via GPC.

### 2.3. Characterization

#### 2.3.1. ^1^H NMR Analysis

A Bruker AVANCE III NMR spectrometer was used to analyze the structure of the polymer in deuterated chloroform (CDCl_3_).

#### 2.3.2. GPC Analysis

The molecular weights and *M*_w_/*M*_n_ (*Ð*) were determined by gel permeation chromatography (GPC) with a Waters 1515 High Pressure Infusion System, WAT038040 Differential Refractive Indicator and Styragel HT5 Columns for THF.

#### 2.3.3. Tensile Tests

Using a laminator, the samples were pressed into 1 mm thick sheets at 150 °C and 10 MPa. The samples were cooled and shaped in cold water. Using a die, the samples were then cut into dumbbell-shaped strips with dimensions of 50 mm × 4 mm × 1 mm after two days of resting. The tensile test was then carried out on an AG-IS (Shimadzu, Kyoto, Japan) universal tensile machine. The test was performed with an SLBL 1 kN tensile force transducer with an accuracy of 0.1 N. The tensile rate for the SIS specimen was 10 cm min^−1^.

#### 2.3.4. DSC Analysis

The fractional profile of the block copolymer was analyzed using a Q100 Differential scanning calorimeter (DSC). Approximately 5 mg of the sample was placed in an aluminum crucible and tested in the range of −70–230 °C at 10 °C min^−1^ after eliminating the thermal history under a nitrogen atmosphere.

#### 2.3.5. AFM Observations

The polymer was dissolved in xylene or chloroform (2 g L^−1^), and the solution was filtered through a 22 μm filter membrane to remove impurities. A drop of sample solution was added to a clean silicon wafer using a 5 mL plastic pipette. It was evaporated naturally for 48 h or leveled by a homogenizer. The polymer imaging was then analyzed using the Tapping mode of the Multimode8 Atomic Force Microscope (AFM), with a tip resonance frequency of 300 kHz and an A/A_0_ equal to 0.6–0.7. In the resulting phase diagram, the PS region was dark, the PI region was basal or bright and the observed individual molecules were bright.

#### 2.3.6. TEM Observations

A 200-mesh copper mesh (carbon support film) was used for sample preparation: the polymer was dissolved in toluene (1 mg mL^−1^), and two to three drops of solution were pipetted onto the liquid surface of a beaker filled with ultrapure water. The drops were retrieved with a copper mesh after a few seconds to obtain an ultrathin polymer layer. The prepared specimens were observed using a Hitachi 7700 transmission electron microscope (TEM).

#### 2.3.7. SAXS Tests

The film sample made in the tensile tests was used in the small-angle X-ray scattering (SAXS) tests with a Xeuss 3.0 Small Angle Scatterometer (Xenocs Inc., Grenoble, France) and a 2500 mm sleeve set.

## 3. Results

**Scheme 1:**
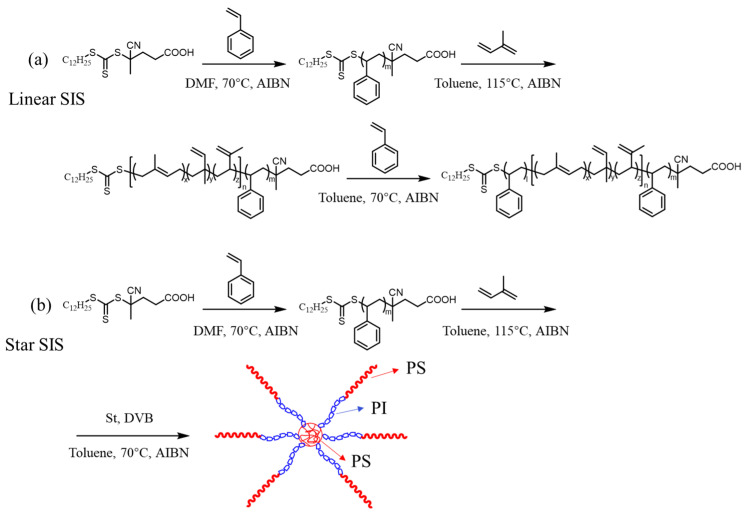
Synthesis process of PS-*b*-PI-*b*-PS: (**a**) linear SIS; (**b**) star SIS.

The synthetic route of preparing SIS linear and star polymers via RAFT polymerization are shown in Scheme 1.

### 3.1. Synthesis of (PS-b-PI) Diblock Copolymer

**Figure 1:**
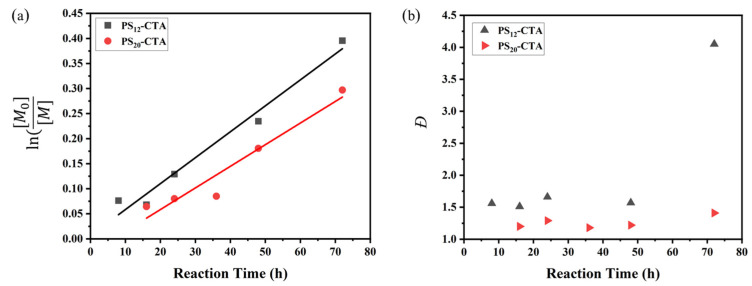
Homopolymerization kinetics of isoprene at 115 °C using PS_12_-CTA or PS_20_-CTA as chain transfer agent: (**a**) kinetic curves; (**b**) molecular weight distribution.

PS-CTA was first synthesized using the small RAFT agent 4-cyano-4-[(dodecylsulfanylthiocarbonyl)sulfanyl] pentanoic acid (DTTCP) (Figure S1). Using PS_12_-CTA and PS_20_-CTA as macro-RAFT agents, we investigated the effect of polymerization time on the homopolymerization of isoprene. The molecular weight and distribution values (*Ð*) are shown in Table S2. The kinetic profile was obtained from the ^1^H NMR in Figure 1, which indicates that the kinetic curve exhibits a straight line, and the majority of the molecular weight distribution is narrow, proving the process control of living polymerization. It can be also clearly observed that the molecular weight of the polymerization group using shorter molecular chains as a macro-RAFT agent increased significantly faster than that of the longer group, which might be due to the inhibitory effect of the chain length of the macromonomer chain transfer agent. The longer the chains were, the less motile the macro-RAFT agent was and thus the longer the RAFT induction period that affected the rate of the chain growth was.

**Scheme 2:**
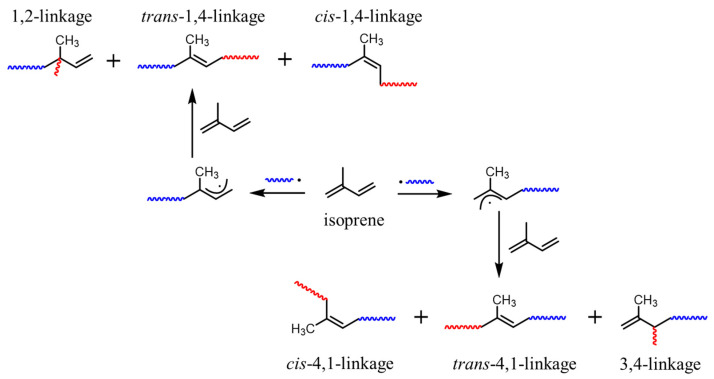
Schematic diagram of possible branching or crosslinking of isoprene during polymerization.

In Table S2, the molecular weights calculated between the ^1^H NMR and GPC showed large differences, indicating that a small amount of crosslinking or transfer reactions might have occurred during the reaction with isoprene itself (Scheme 2), which may have resulted in dead chains [53].

### 3.2. Synthesis of SIS Linear and Star Triblock Copolymers

#### 3.2.1. Influence Factors of Star-Forming Reaction

In order to determine the better polymerization condition of the star-forming reaction, various factors were examined in the arm-first process, including the polymerization time, the solid content of PS-*b*-PI, the monomer and crosslinker ratio, the types of crosslinker and solvent and the chain extension temperature of the PS-*b*-PI. The relevant experimental factors are summarized in Table S1, and the product component was characterized by GPC and is shown in Figure S2. In the table and figure, “gel” represents a polymerization failure, while “15m-18 (36S)” represents that the solid content of PS-*b*-PI was 0.015 mol/L and the feeding ratio of [DVB]:[St]:[PS-*b*-PI] was 18:36:1, where “s” represents styrene. When the solid content and reaction time were increased from 0.015 mol L^−1^, 48 h to 0.020 mol L^−1^, 72 h, the star-forming conversion was not significantly improved. The increase in the crosslinker and monomer ratio helped to increase the conversion rate; however, if the dosage was too high, it would cause the crosslinker molecules to have an excessive conversion rate, which would induce the gelation of the system [51]. The synthesis of PS-*b*-PI often requires high reaction temperatures in order to achieve significant isoprene conversions during block copolymerization. However, compared with chain expansion at 115 °C, the conversion rate of the arm-first star-forming reaction using PS-*b*-PI was obtained at 125 °C as the arm molecule decreased from 70% to 20% due to the high-temperature reaction at above 120 °C during the homopolymerization of isoprene, which was damaging to the end group of the RAFT agent [53]. In conclusion, the star SIS was synthesized with a solid content of 0.015 mol L^−1^ in 48 h, and the dosage of the crosslinker and monomer was [DVB]:[St]:[PS-*b*-PI] = 24:36:1. We also used the arm molecule derived from the isoprene chain extension at 115 °C for the star-forming reaction.

#### 3.2.2. Characterization of the Molecular Structure of SIS

**Figure 2:**
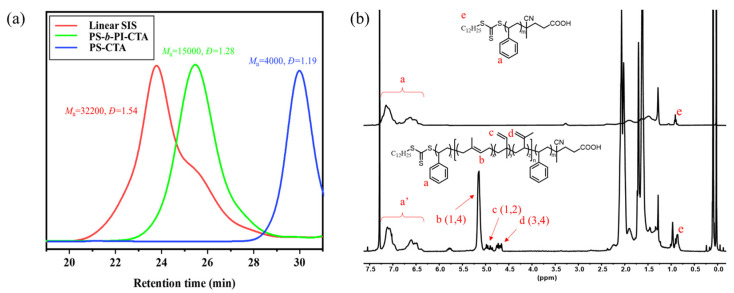
Molecular weight and structure of PS-CTA, PS-*b*-PI-CTA and linear SISs; (**a**) GPC traces; (**b**) ^1^H NMR spectra.

Using PS-*b*-PI a macro-RAFT agent and a molecular arm, linear and star SISs were prepared with the improved polymerization condition as discussed above, and the molecular weight information is summarized in Table 1 and Figure 2. During the synthesis of the linear SIS, the monomer ratio was slightly increased compared to the synthesis of PS-CTA. Overall, a series of linear triblock copolymers whose hard segment contents (wt% of PS) ranged from 42 to 80% were prepared well. Notably, the molecular weight growth of the star-shaped polymers does not exhibit a strong regularity. For example, in the L6 group, the feeding ratio was [PS-*b*-PI-CTA]:[St] = 1:150, but the polystyrene only had a degree of polymerization of 50 after 48 h with an arm conversion of about 33% due to the high molecular weight, which limited the movement of the chain segment, and the high temperature reaction in the synthesis of PS-*b*-PI. As shown in Table 1, the star-shaped polymers synthesized in this work covered molecular weights (*M*_n_) ranging from 70,300 to 168,200 and hard segment contents (wt% of PS) ranging from 21 to 57 for subsequent research. Figure 2 shows that with the synthesis of the block polymer, the molecular weight increased gradually, and the 1,4 structure occupied the majority of the PI, which proved the success of the synthesis of the linear SIS. According to some studies on the end groups of RAFT agents, the change in the end group of RAFT agents to the thiol group requires ammonolysis in the presence of tributylphosphine [54,55,56], whereas the trithione end groups remained relatively stable when only monomers and initiators were added in our experiments, which helped achieve the successful SIS synthesis. Figure 2a also exhibits that there is a shoulder peak observed in the GPC results of the SIS representing dead chains that resulted from the polymerization of isoprene in the previous step due to a side reaction (Scheme 2). These dead chains lack trithione end groups and do not participate in subsequent reactions. Figure 2b presents the typical ^1^H NMR spectra of PS-CTA and linear SIS. The dodecyl terminal methyl peak of the RAFT agent is represented by “e” (δ = 0.88 ppm), while “b”, “c”, and “d” correspond to the isoprene units of structures 1,4, 3,4, and 1,2, respectively. The peak “a” corresponds to the overall peak of the benzene ring of styrene. It is observed that there is no significant overlap in the spectra, indicating the reliability of the NMR data.

### 3.3. Characterization of SIS Linear and Star Triblock Copolymers

#### 3.3.1. Thermal and Mechanical Properties

**Figure 3:**
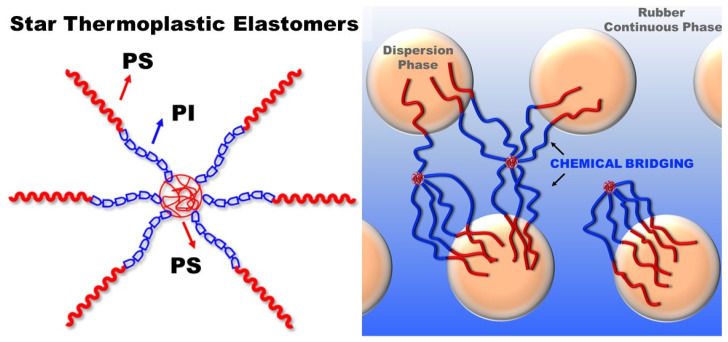
Schematic diagram of chemical bridging theory of star thermoplastic elastomer.

As shown in Figure 3, the properties of the thermoplastic elastomers originate from the partitioning of phases. From the bridging theory, the interactions between the phase regions originate from bridges. If the ends of the triblock molecular chain are located in different phase regions, they are able to connect different dispersed phases, providing them with the effect of physical crosslinking. The single molecule of the star structure has the ability to connect multiple phase regions, thus increasing the crosslink density to a certain extent and enhancing the tensile properties of the material.

**Figure 4:**
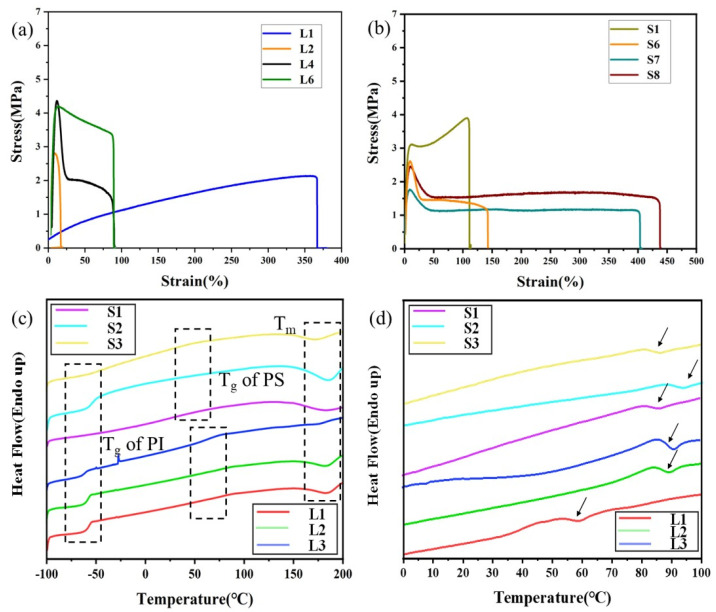
Stress–strain (**a**,**b**) and DSC (**c**,**d**) curves of linear SISs and star SISs.

In thermoplastic elastomers, the ratio of hard segment (PS) has a greater influence on the material properties than other factors. Figure 4a,b show the stress–strain curves of a well-prepared SIS. The tensile curves of the linear polymers exhibited an overall trend of an increasing tensile strength and decreasing elongation at break as the content of the hard segment increased from 42% in L1 to 51% in L2. A similar pattern was also observed in the tensile curves of the star polymers. The sample of L4 showed a significant necking phenomenon with a stress drop and an increase in strain, perhaps due to defects such as air bubbles or cracks during sample preparation. However, if the hard segment content was over 48% it would make the sample brittle and even unable to be pressed into sheets, such as L3, L5 and S2–S5. For this reason, the tensile behavior of L2 exhibited brittle deformation due to the fact that the styrene content in L2 was close to the limit content of the elastomer hard segment. Meanwhile, the polymers with a star structure did not show substantially enhanced mechanical properties in this work, probably because of the higher number of arms of the star molecules in this work, which resulted in a reduction in the phasing ability. DSC heating curves of the SIS in Figure 4c indicate that the shape of the spectral lines is less affected by the components: the T_g_ of the PI section was about −58 °C, and the glass transition temperature was relatively low and insignificant due to the short design chain length of the PS section. In addition, all samples exhibited a melting temperature of about 170 °C. Both the linear and star samples in the cooling curves (Figure 4d) show a significant heat absorption of around 90 °C, which might correspond to the reaction of the RAFT agent end groups.

#### 3.3.2. Morphology

**Figure 5:**
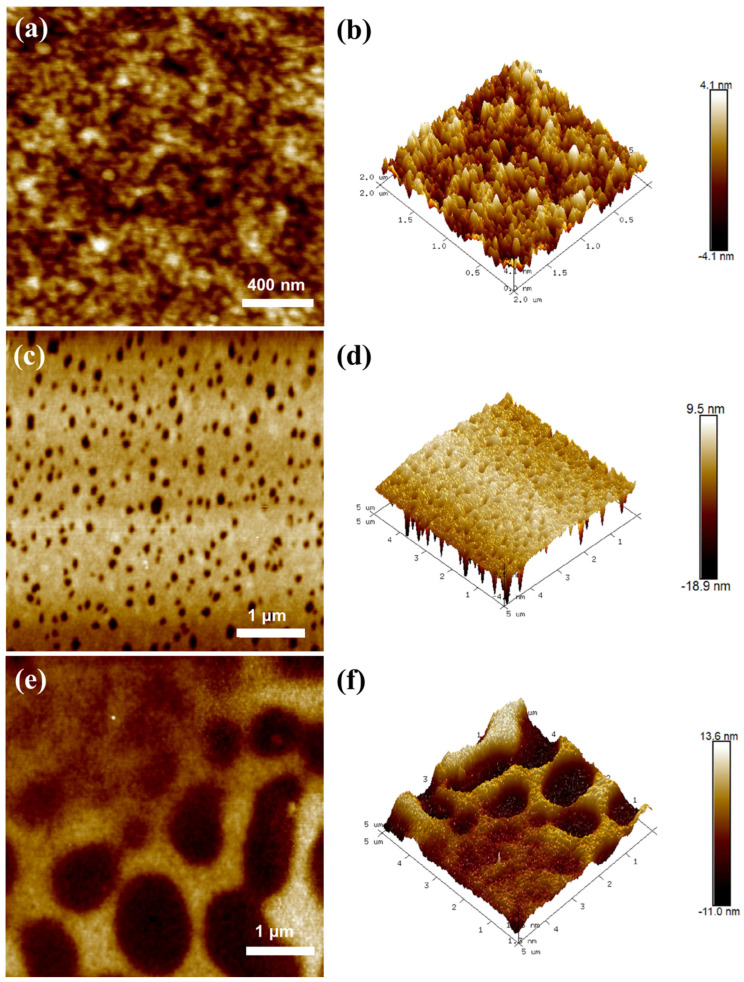
Atomic force microscope images and 3D height diagram of linear SISs (film-dried naturally). L1: (**a**,**b**); L2: (**c**,**d**); L3: (**e**,**f**).

Block copolymers formed by styrene with butadiene and isoprene will spontaneously exhibit topographic composition phase phenomenon in the polymer matrix state which can be directly observed by AFM, SFM and SEM [57]. It is known from the linear SIS AFM images obtained by the natural volatile drying method of film formation using xylene as the solvent (Figure 5) that L1 (Figure 5a,b) exhibits a compact phase separation structure because of its higher molecular weight. L2 (Figure 5c,d) shows a typical spherical hard segment morphology, and the higher hard segment content also resulted in larger phase domains, possibly forming larger phase domains (Figure 5e,f). When using chloroform as the solvent and spin-coating the film with a homogenizer, the AFM diagram shows a bicontinuous phase structure and a significant reduction in the size of phase domains (Figure S3). Similar conclusions could be drawn from the AFM phase images and TEM images in Figures S4 and S5 as well, which prove that the linear SIS has a strong microscopic phase separation capability.

**Figure 6:**
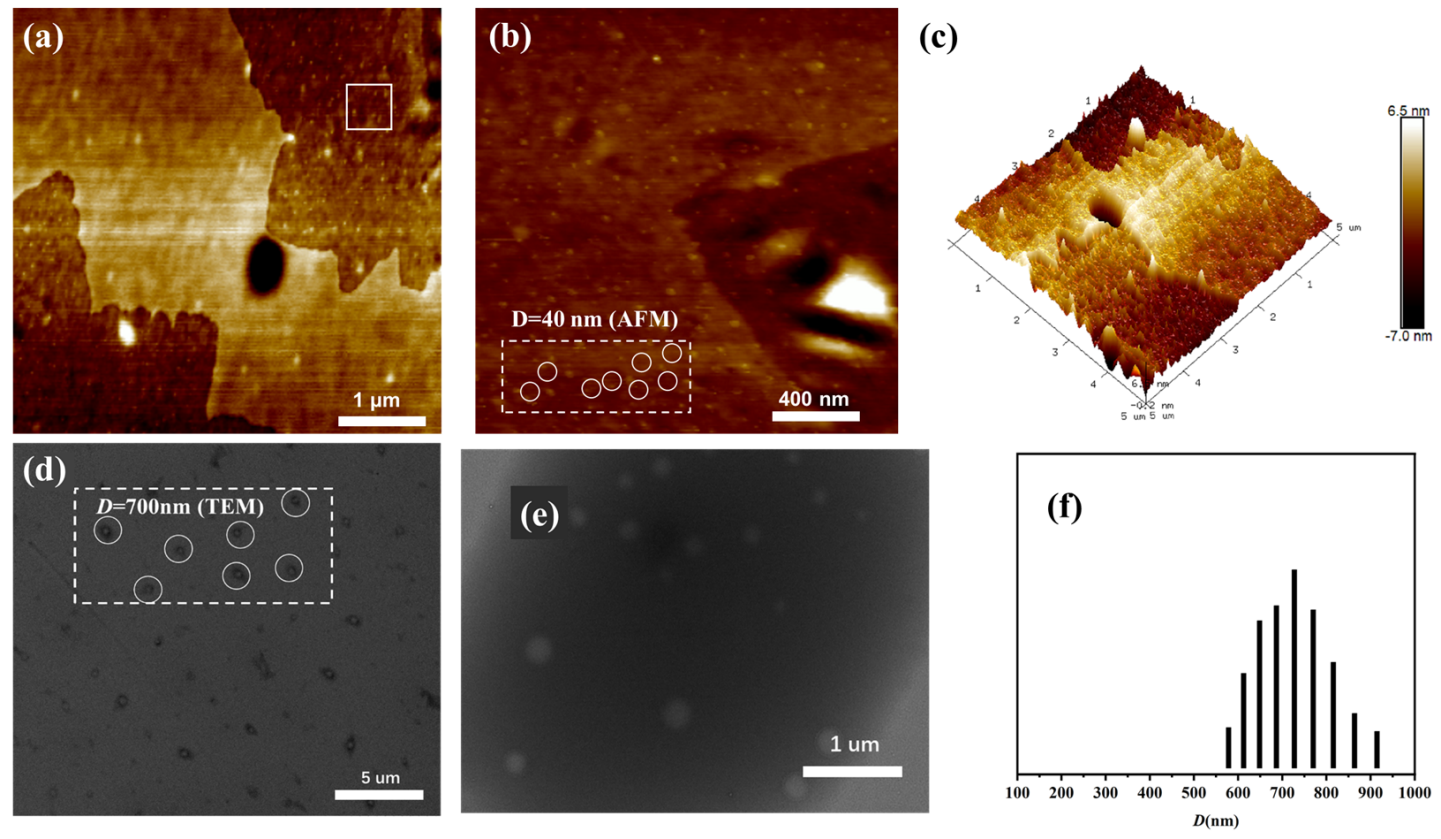
AFM images (**a**–**c**) and TEM images (**d**–**f**) of S5: (**a**,**b**) AFM images under different microscale; (**c**) 3D height diagram; (**d**) TEM images; (**e**) microphase separation in agglomerated particles; (**f**) particle size distribution from DLS.

Regarding the microscopic shape of the star SIS in this work (Figure 6), the AFM images (Figure 6a–c and Figure S6) show no obvious phase separation morphology, but a structure similar to that of free particles indicates that the presence of the star structure affected the ability to split the phase. The same nanoparticle morphology was observed in the TEM images, and the presence of nanoparticles of various scales in the system was illustrated in the DLS (Figure 6d–f). If the isoprene soft segment content was too high, the core region might be completely encapsulated in it, making it difficult to observe the non-discolored core region (Figure 6e).

**Figure 7:**
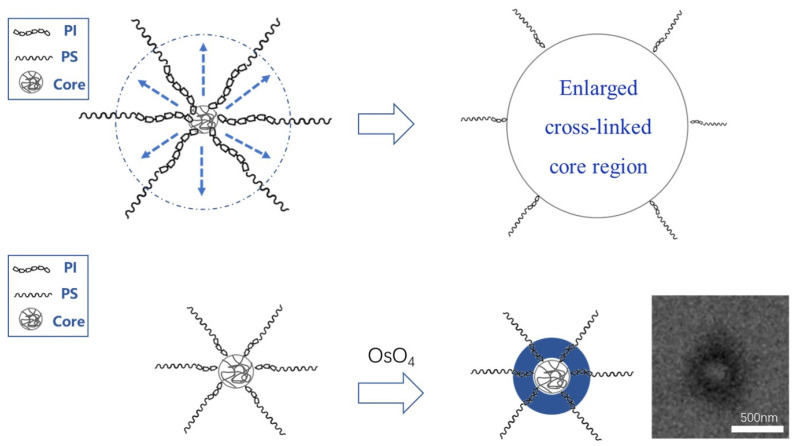
Formation mechanism of nanoparticles and hollow nanoparticles.

The presence of hollow nanoparticles can also be observed in the figure, which can be explained in Figure 7. For a normal core crosslinked star polymer, each arm contributes about 30% to the formation of the core region. However, in this work, isoprene was involved in the polymerization and the arm molecules possessed isolated double bonds; thus, the crosslinker monomer intentionally expanded the crosslinked region during the star-forming reaction, making the original core region much larger and eventually making it exhibit nanoparticle properties. Meanwhile, osmium tetroxide dye is a double-bond-specific dye, and only the isoprene soft segment could be greatly stained in the crosslinked particles, while the core region consisting of styrene and DVB was left unstained or lightly stained. Therefore, the system exhibited the microscopic morphology of hollow nanoparticles.

**Figure 8:**
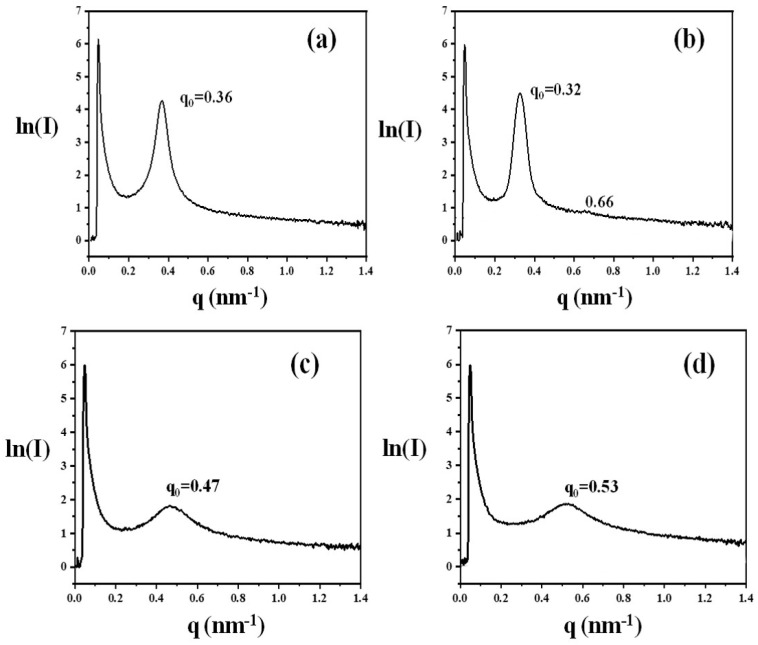
SAXS results of SISs: (**a**) L1; (**b**) L2; (**c**) S1; (**d**) S2.

To better illustrate the phase separation structure of the SIS, linear and star-shaped SISs with similar hard segment ratios were selected in this work for a comparative study of SAXS in Figure 8. The scattering peaks at 0.3 nm^−1^ indicate that the linear system still had a strong phase separation ability, and the calculated phase domains were 17 nm and 19 nm for L1 and L2, respectively, from the equation 2π/d = q. The corresponding scattering peaks in the star group were shifted to the right and the peak shape became wider, indicating that the scale of the sequence structure became smaller, and the interface of phase separation tended to be blurred, proving that the star SISs had a weaker phase-splitting ability.

## 4. Conclusions

In this paper, linear and star SIS thermoplastic elastomers were prepared via RAFT polymerization, and their thermomechanical properties and micromorphology were investigated. Firstly, in the preparation of the diblock copolymer PS-*b*-PI, the RAFT solution polymerization method is still applicable to the living control process of the low-activity monomer isoprene, and the degree of the polymerization can be effectively increased by increasing the temperature, which should be noted because of the temperature effect on the end group of the RAFT agents. We first prepared a series of star SISs via the arm-first strategy with PS-*b*-PI as the arm and improved the star-forming conversion rate by controlling the influencing factors in the reaction. We verified the experimental results by GPC. It was observed that the best arm conversion rate during the star-formation process was achieved when the solid content of PS-*b*-PI was 0.015 mol/L and the dosage of crosslinker and monomer (styrene) was [DVB]:[St]:[PS-*b*-PI] = 24:36:1 with a polymerization time of 48 h. Next, the star and linear SISs prepared under the above optimal experimental conditions were characterized via tensile tests and DSC, and it was found that increasing the hard segment content help improved the tensile strength but led to a significant decrease in material toughness. Finally, the microstructure was characterized by AFM, TEM and SAXS, which proved that with the morphology of phase separation, SIS has the characteristics of thermoplastic elastomers, and the synthesized star SIS exists in the form of spherical nanoparticles. This has enriched the synthesis system of thermoplastic elastomers, broadened the application scope of nuclear crosslinked star polymers and provided new ideas for the synthesis of polymer nanoparticles.

## Figures and Tables

**Table 1:** Characteristics of SIS linear and star triblock copolymers.

Entry	Polymer	wt%PS	*M*_n, NMR_ ^1^	*M*_n, GPC_ ^2^	*Ð* ^2^	Ratio of PI (1,4) ^1^	Ratio of PI (1,2) ^1^	Ratio of PI (3,4) ^1^
L1	PS_40_-*b*-PI_132_-*b*-PS_23_	42	16,000	37,000	3.68	85	7	8
L2	PS_22_-*b*-PI_70_-*b*-PS_24_	51	10,000	51,800	1.75	87	7	6
L3	PS_22_-*b*-PI_20_-*b*-PS_30_	80	7200	32,200	1.54	88	6	6
L4	PS_23_-*b*-PI_84_-*b*-PS_30_	49	11,600	22,000	1.57	87	6	7
L5	PS_23_-*b*-PI_84_-*b*-PS_60_	60	14,800	45,000	2.23	90	5	5
L6	PS_40_-*b*-PI_161_-*b*-PS_50_	46	20,700	33,200	2.20	89	5	6
S1	star-(PS_22_-*b*-PI_46_)_30_	42	-	162,900	1.47	88	5	6
S2	star-(PS_25_-*b*-PI_39_)_13_	50	-	70,300	1.32	84	7	9
S3	star-(PS_13_-*b*-PI_16_)_40_	55	-	107,500	3.36	80	12	8
S4	star-(PS_20_-*b*-PI_25_)_40_	55	-	168,200	2.34	85	9	4
S5	star-(PS_34_-*b*-PI_39_)_16_	57	-	104,600	1.43	89	5	6
S6	star-(PS_23_-*b*-PI_73_)_19_	33	-	145,700	2.06	85	7	8
S7	star-(PS_23_-*b*-PI_103_)_14_	25	-	135,600	1.77	89	6	5
S8	star-(PS_23_-*b*-PI_132_)_9_	21	-	100,500	2.35	82	8	10

^1^ Calculated by 1H NMR. ^2^ Molecular weight and polydispersity were determined by THF GPC.

## Data Availability

The data presented in this study are available on request from the corresponding author.

## Supplementary Materials

The following supporting information can be downloaded at: www.mdpi.com/article/10.3390/polym15092002/s1, Figure S1: ^1^H NMR spectra of RAFT agent DTTCP; Figure S2: Various factors on the process of arm-first: (a) polymerization time, (b) solid content, (c) monomer ratio, (d) solvent, (e) crosslinker, (f) chain extension temperature; Figure S3: Atomic force microscope images and 3D height diagram of linear SIS (spin-coating). L1: (a,b); L2: (c,d); Figure S4: Atomic force microscope phase images of linear SIS prepared (film natural drying). L1: (a); L2: (b); Figure S5: TEM images of L1; Figure S6: AFM phase images of S5, (a,b) are corresponding to (a,b) in Figure 6; Table S1: Various factors on the process of arm-first; Table S2: PI obtained by various reaction condition.
