# Supplementary material for: Star-Shaped Thermoplastic Elastomers Prepared via RAFT Polymerization
Source: Polymers (Basel). 2023 Apr 23;15(9):2002. doi: 10.3390/polym15092002 (PMC10180775; doi:10.3390/polym15092002)
Supplement: Supplementary file 1 [file polymers-15-02002-s001.zip › polymers-2246784-supplementary.pdf]

Supporting information

Star–shaped thermoplastic elastomers prepared by RAFT polymerization

Hao Ge [1, 2], Wencheng Shi [1, 2], Chen He[3], Anchao Feng [1, 2]* and San H. Thang[4]

1 State Key Laboratory of Organic-Inorganic Composites, Beijing University of Chemical Technology, Beijing 100029, China;

2 Center of Advanced Elastomer Materials, College of Materials Science and Engineering, Beijing University of Chemical Technology, Beijing 100029, China;

3 Aerospace Research Institute of Materials & Processing Technology, Beijing, 100076, China;

4 School of Chemistry, Monash University, Clayton, Victoria 3800, Australia;

* Correspondence: fengac@mail.buct.edu.cn;

Synthesis of small RAFT agent DTTCP

NaH (3.15g, 79mmol) and 150mL of anhydrous ethyl ether were introduced to a 250mL spherical Schlenk flask, which was placed in 0ºC water bath. After stirring the solution to a uniform off-white suspension, dodecyl mercaptan (15.4g, 76mmol) and carbon disulfide (CS_2, 6.0g, 79mmol) were added into then flask with a dropping funnel. After 3h of reaction below 10ºC, the yellow paste product was extracted filtered and dried in a vacuum drying oven at 30ºC.

The above yellow product (14.72g, 49mol) and 100mL of anhydrous ethyl ether was taken in a 250 mL flask and solid iodine is added in batches during stirring. After 1h of reaction, the mixture was transferred to a 500mL partition funnel and wash with saturated sodium thiosulfate ($Na_2S_2O_3$) solution to remove unreacted iodine monomers and the resulting white precipitate sodium iodide (NaI). Spin evaporation to remove excess solvent and got orange-red solid.

The above product (2.76g, 5mmol), 4,4'-Azobis(4-cyanovalericacid) (ACVA, 2.10g, 7.5mmol) and 50mL of ethyl acetate were added in a 250 mL flask and reflux the flask at 90-100ºC for 18h. After removing the solvent by rotary evaporation, introduced 500 mL of hexane and the system was refined and recrystallized at -20ºC. Finally, a light yellow powder solid is obtained, which was analyzed by ^{1}H NMR.

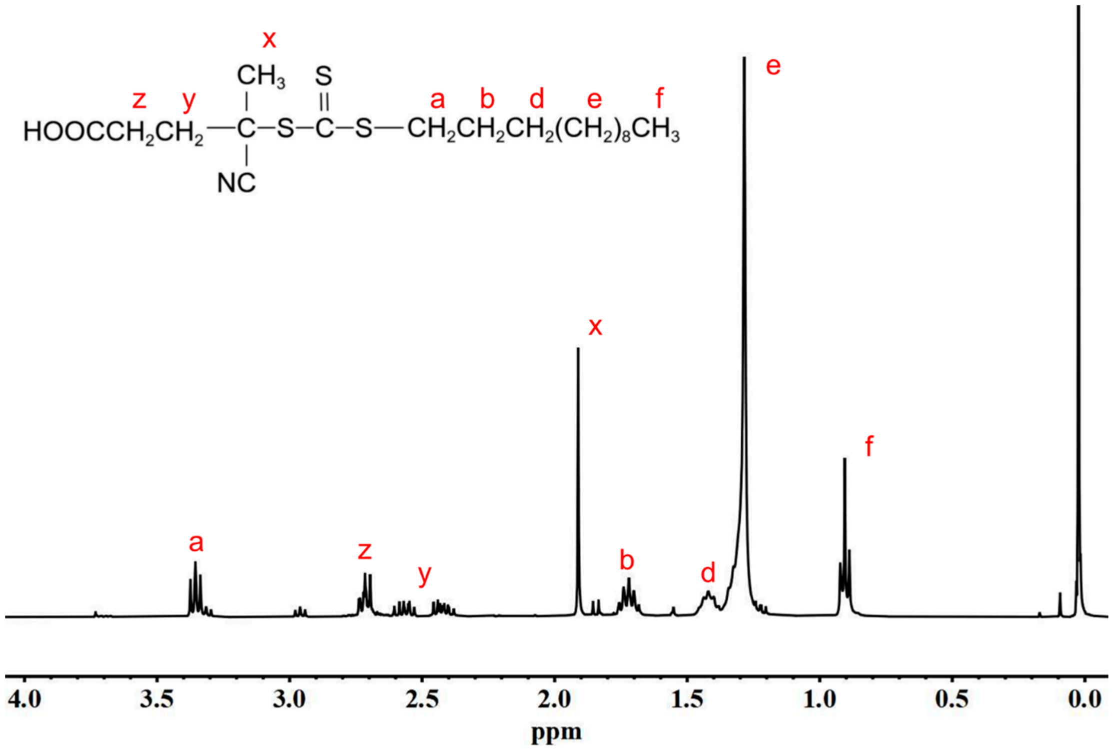

Figure S1. ^{1}H NMR spectra of RAFT agent DTTCP.

Table S1. Various factors on the process of arm-first

Group	Entry	[MI]	arm	[crosslinker] /[MI]	[styrene] /[MI]	solvent	t/h	gel
	1				-		24	-
	2			12	-		36	-
	3				-		48	-
	4				-		24	-
	5	0.006		18	-		36	-
	6				-		48	-
I	7		PSt_{40}-*b*-PI_{132}		-	toluene	24	-
	8			24	-		36	-
	9				-		48	-
	10				-		24	-
	11				-		36	-
	12	0.015		12	-		48	-
	13				-		72	-
	14			24	-		48	-

	15				-		72	-
	16			36	-		48	gel
	17				-		72	gel
	18			48	-		48	gel
	19				-		72	gel
	20	0.02		24	-		48	-
	21				-		72	-
II	22				12			-
	23				24			-
	24	0.015			36			-
	25			18	48			gel
	26				60			gel
	27	0.006	PSt_{40}-*b*-PI_{132}		36	toluene	48	-
	28				24			-
	29	0.015						-
	30				36			-
	31	0.02		24				-
	32	0.035						gel
III	33					toluene/ DMF=9/1		gel
	34	0.015	PSt_{40}-*b*-PI_{132}	18	36	toluene/ DMF=8/2		gel
	35					toluene/ DMF=7/3		-
	36					toluene/ DMF=6/4		-
IV	37			36 (EDGMA)				-
	38	0.015	PSt_{40}-*b*-PI_{132}	24 (EDGMA)	36	toluene		-
	39			12 (EDGMA)				-
V	40	0.015	PSt_{22}-*b*-PI_{70}	24	36	toluene		-

	41	0.025	115°C	gel
	42	0.035		gel
	43	0.015		-
	44	0.025	PSt_{22}-*b*-PI_{66} 125°C	gel
	45	0.035		gel

Table S2. PI obtained by various reaction condition.

Entry	CTA	Time (h)	Temperature (°C)	$M_{n,NMR}$ [a] (g mol^{-1})	$M_{n,GPC}$ [b] (g mol^{-1})	$Đ$ [b]
1	PS_{12}-CTA	8	115°C	3600	8500	1.56
2	PS_{12}-CTA	16	115°C	3300	8700	1.51
3	PS_{12}-CTA	24	115°C	4900	12700	1.66
4	PS_{12}-CTA	48	115°C	7300	28700	1.57
5	PS_{12}-CTA	72	115°C	10500	42700	4.05
6	PS_{12}-CTA	16	115°C	4200	8400	1.20
7	PS_{12}-CTA	24	115°C	4600	9700	1.29
8	PS_{12}-CTA	36	115°C	4700	9900	1.18
9	PS_{12}-CTA	48	115°C	7000	13800	1.22
10	PS_{12}-CTA	72	115°C	9500	11000	1.41

[a]Molecular weight were calculated by ^{1}H NMR. [b]Molecular weight and polydispersity were determined by THF GPC.

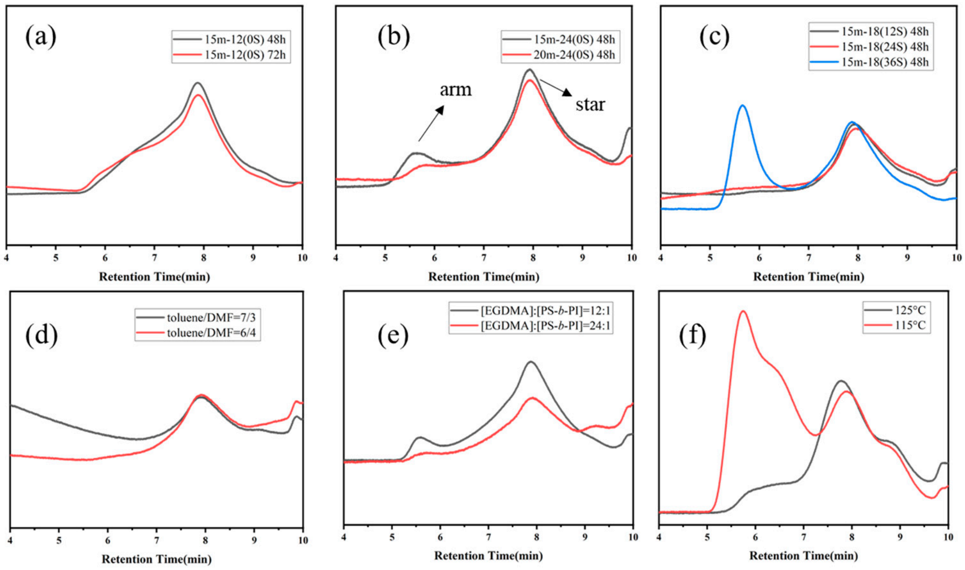

Figure S2. Various factors on the process of arm-first: a) polymerization time, b) solid content, c) monomer ratio, d) solvent, e) crosslinker, f) chain extension temperature.

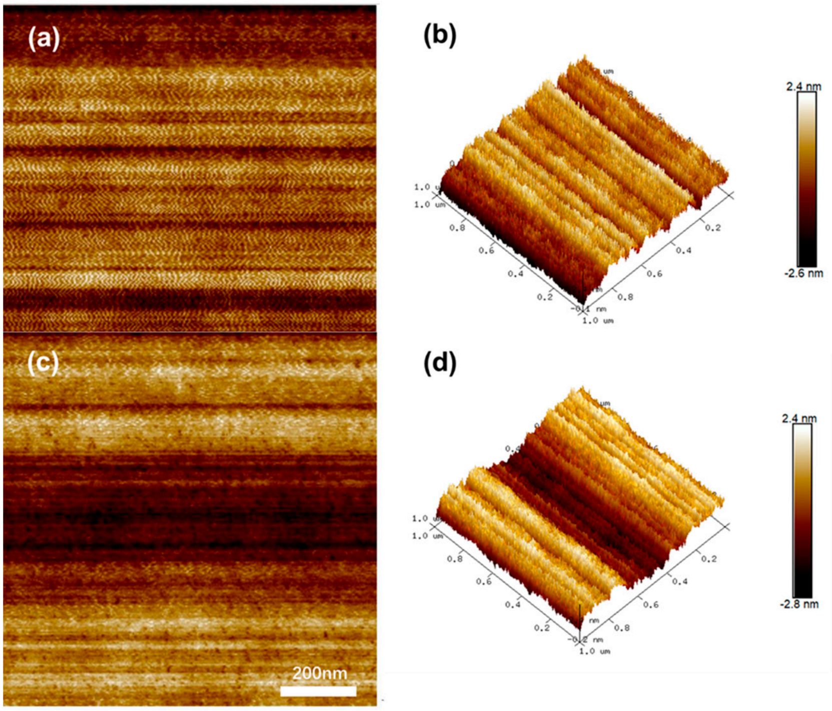

Figure S3. Atomic force microscope images and 3D height diagram of linear SIS (spin-coating). L1: (a), (b); L2: (c), (d).

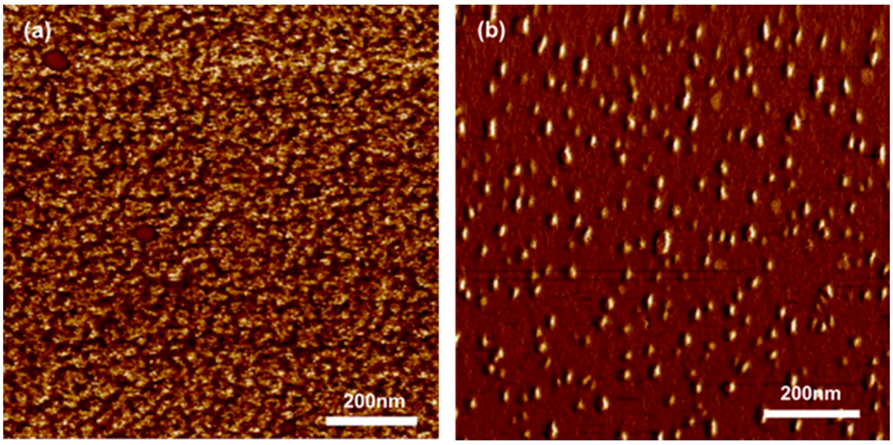

Figure S4. Atomic force microscope phase images of linear SIS prepared (film natural drying). L1: (a); L2: (b).

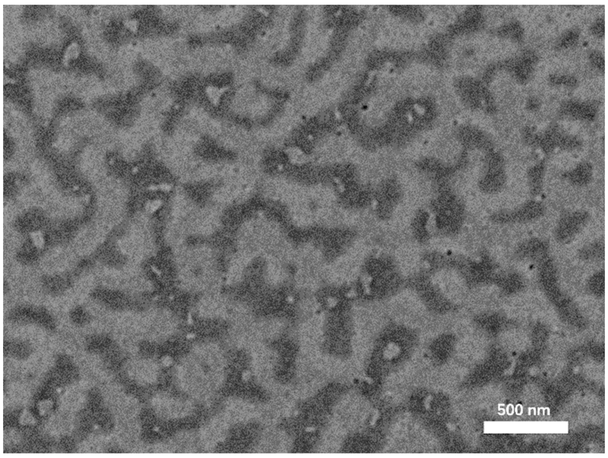

Figure S5. TEM images of L1.

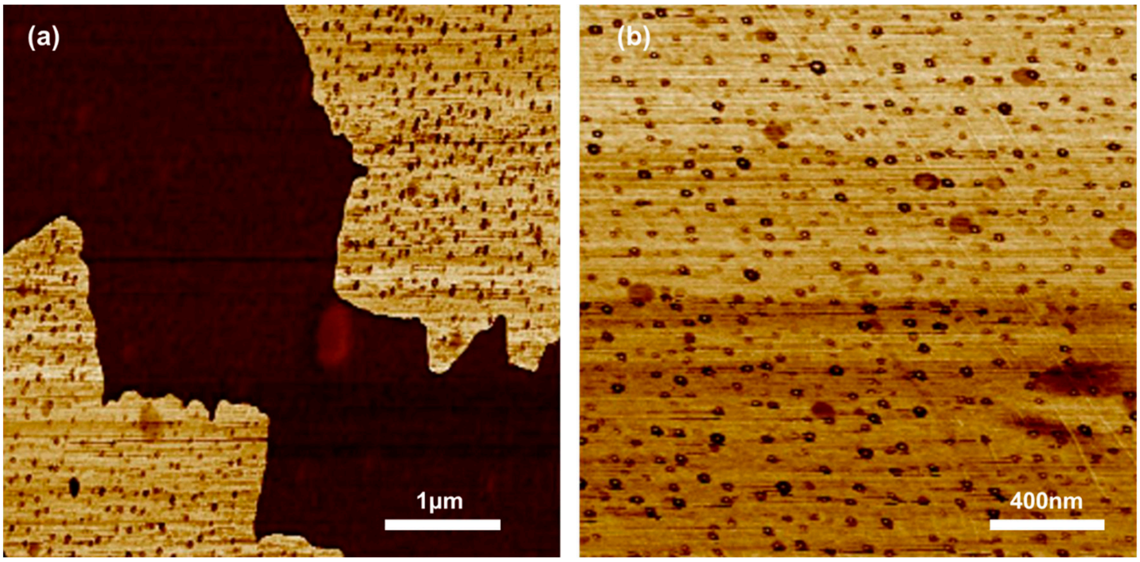

Figure S6. AFM phase images of S5, (a) and (b) are corresponding to (a) and (b) in Figure 6..
